# Physiological and Biochemical Vascular Reactivity Parameters of Angiotensin II and the Action of Biased Agonist TRV023

**DOI:** 10.1155/2020/3092721

**Published:** 2020-02-21

**Authors:** Marcos André Soares Leal, Thanisia de Almeida, João Guilherme Torres, Luciene Cristina Gastalho Campos, Elisardo Corral Vasquez, Valério Garrone Barauna

**Affiliations:** ^1^Laboratory of Translational Physiology, Federal University of Espirito, Vitória, Espirito Santo, Brazil; ^2^Laboratory of Molecular Physiology, Federal University of Espírito Santo, Vitória, Espirito Santo, Brazil; ^3^Postgraduate Program in Biochemistry, Federal University of the Pampa, Uruguaiana, RS, Brazil; ^4^Department of Biological Science, Santa Cruz State University, Ilheus, BA, Brazil

## Abstract

Vascular reactivity experiments using isolated aortic rings have been widely used as a model for physiological and pharmacological studies since the early sixties. Here, we suggest several parameters that the researcher should pay attention to when investigating angiotensin II in their experimental models. Angiotensin II is one of the active peptides of the renin-angiotensin system and exerts its effect through the AT1 and AT2 receptors. Some studies seek to understand the effects of angiotensin II receptors at the vascular level by using vascular reactivity experiments. However, because of the large number of variations, there are only a handful of reactivity studies that seek to use this method. Thus, the objective of this study was to standardize experimental methods with angiotensin II, through vascular reactivity protocols. For this, variables such as basal tension, concentration interval, single concentration, curve concentration response, and multiple experiments using the same aortic ring were developed using the technique of vascular reactivity in an organ bath. This is the first study that has standardized the vascular reactivity protocol. In addition, we demonstrated the effects of TRV023-biased ligand of the AT1R at vascular sites.

## 1. Introduction

The renin-angiotensin system (RAS) is critical in maintaining homeostasis of the cardiovascular system [1]. In the classical RAS axis, juxtaglomerular cells of the kidney release renin which catalyzes angiotensinogen cleavage to form the glycopeptide angiotensin I. Angiotensin I is converted to angiotensin II by the angiotensin-converting enzyme (ACE) which removes the two terminal peptides from angiotensin I. Angiotensin II (Ang II) is the main peptide formed in this classical RAS axis and binds to angiotensin type-1 receptor (AT1R) and angiotensin type-2 receptor (AT2R) [2].

The binding of Ang II to AT1R can cause detrimental effects such as vasoconstriction and proliferation of vascular smooth muscles and endothelial dysfunction. For instance, the binding of Ang II to AT1R impairs endothelial function by increasing NAD(P)H oxidase activity and producing superoxide anions [3]. Overall, these processes can contribute to the development of cardiovascular disease.

AT1Rs are classically G-protein-coupled receptors and exert their signaling through G-protein dependent and independent pathways. The AT1R-biased ligand simultaneously competitively antagonizes Ang II-stimulated G-protein signaling while stimulating the *β*-arrestin pathway [4]. This approach has been shown to block the detrimental effects of Ang II such as vasoconstriction and cardiac hypertrophy, while enhancing beneficial effects on myocardial contractility, and activation of antiapoptotic pathways. Although AT1R-biased ligands have already been tested in clinical trials, the mechanisms of action have only been demonstrated in heart and kidney tissues [5–8]. The direct effects in the vascular tissue have never been studied.

There are plenty of studies using different protocols of vascular reactivity to Ang II [9–12]. The majority of studies found in the literature only indirectly investigated the role of Ang II performing vascular reactivity curves under the blockade of AT1R [13, 14]. However, these studies have been shown to produce a lot of variations in their results. The importance of studying vascular reactivity of peptides from the RAS was also addressed by Lautner et al. who described technique and pitfalls about the use of angiotensin-(1–7) standard protocols for Ang 1–7 [15].

Because of the difficult standardization and a wide variety of existing angiotensin II vascular contraction protocols, the aim of the present study was to standardize ideal conditions for Ang II vascular reactivity in aortic rings using temporal, baseline tension, and angiotensin concentration parameters. In addition, we tested the effects of TRV023 in aortic rings, a peptide with biased agonist activity to the AT1 receptor. TRV023 activates the *β*-arrestin pathway instead of the G-protein, and it was used in this study in order to verify *β*-arrestin vascular effects *in vitro*.

## 2. Material and Methods

### 2.1. Animals

Twelve-week-old male Wistar rats (*Rattus norvegicus albinus*) were obtained from the Federal University of Espirito Santo central animal facility, Brazil. Rats were kept in groups of five in plastic cages with controlled temperature (22-23°C), light-dark cycle of 12 : 12-h, and with free access to food and water. A total of 32 animals were used. All protocols and surgical procedures used were in accordance with the guidelines of the Brazilian College for Animal Experimentation and were approved by the Ethics Committee of the Federal University of Espirito Santo (CEUA-UFES, Protocol 011/2014).

### 2.2. Vascular Reactivity Experiments

Vascular reactivity experiments were performed as described previously [16–18]. Rats were euthanized by decapitation after an overdose of sodium thiopental (Cristalia, Sao Paulo, Brazil, 200 mg/kg, i.p.), and the thoracic aorta was carefully dissected out and cleaned of fat and connective tissue. For reactivity experiments, the aorta was divided into cylindrical segments of 2 mm in length. To investigate the role of the angiotensin II on the smooth muscle layer, all rings had their endothelium removed mechanically by rubbing the lumen with a needle.

Segments of the thoracic aorta were mounted in an organ bath with Krebs–Henseleit solution (in mM: NaCl 118, KCl 4.7, NaHCO_3_ 23, CaCl_2_-2H_2_O 2.5, KH_2_PO_4_ 1.2, MgSO_4_-7H_2_O 1.2, glucose 11, and EDTA 0.01) at a temperature of 37°C gassed with 95% O_2_ and 5% CO_2_ (pH 7.4) and kept at constant baseline tension (1 g, 2 g, or 3 g). Isometric tension was recorded using an isometric force transducer (TSD125C, CA, U.S.A) attached to an acquisition system (MP100, BIOPAC System, Inc., Santa Barbara, CA, U.S.A) and connected to a computer. All aortic rings were exposed twice to 75 mM KCl (30 min) to check the functional integrity of the vessel, as well as the maximum developed tension. The endothelium removal was confirmed by the inability of acetylcholine to induce relaxation greater than 10% of the previous contraction to phenylephrine (Phe). The muscular layer integrity was confirmed if the aortic ring contract at least 2 g when KCl was applied.

### 2.3. Cumulative Concentration-Effect Curve (CCEC) to Angiotensin II

To study the optimal interval time to perform a CCEC to angiotensin II, aortic rings were exposed to Ang II (1nM to 10 mM) with 15, 30, 60, and 120 seconds of interval between each concentration application.

### 2.4. NonnCumulative Concentration-Effect Curve (NCCEC) to Angiotensin II

Aortic contraction to Ang II was performed with a single concentration of the agonist in the separated aortic ring. After an equilibration period, each aortic ring was exposed to a single concentration of Ang II (1 nM to 10 mM). The maximal contraction was considered when a plateau was reached, and the tension starts going back to baseline.

### 2.5. Determination of Optimal Resting Tension

To study the optimal resting tension to perform vascular reactivity experiments to Ang II, aortic rings were equilibrated for 45 min period with 1 g, 2 g, or 3 g of resting tension. Afterwards, aortic rings were exposed to CCEC of Ang II.

### 2.6. Evaluation of Repetitive CCEC versus NCCEC to Angiotensin II

To study the possibility of using the same aortic tissue twice for vascular reactivity experiments, after the last concentration of the agonist has been added and the effect obtained, the tissues were rinsed several times until return to baseline and let another 60 min equilibration period until performing another experiment (CCEC or NCCEC).

## 3. Western Blot

Aortic rings were stimulated with Angiotensin II or TRV023 for 10 minutes and then were homogenized in RIPA buffer (50 mm Tris (pH 7.4), 0.5% Nonidet P-40, 0.2% sodium deoxycholate, 100 mm NaCl, 1 mm EGTA, 1 mm phenylmethylsulfonyl fluoride, 1 *μ*g/ml aprotinin, 1 mm sodium orthovanadate, and 1 mm NaF). Insoluble tissues were removed by centrifugation at 3,000×g and 4°C for 10 min. Samples were loaded onto polyacrylamide gels (15%) and subjected to SDS-PAGE. After electrophoresis, proteins were electrotransferred to nitrocellulose membranes (BioRad Biosciences, USA). The membrane was then incubated in a blocking buffer (5% BSA, 10 mM Tris-HCl (pH 7.6), 150 mM NaCl, and 0.1% Tween 20) for 2 h at room temperature and then incubated overnight at 4°C with rabbit anti-MLC and anti-p-MLC antibodies. Binding of the primary antibody was detected with the use of specific peroxidase-conjugated secondary antibodies, and enhanced chemiluminescence reagents (Amersham Biosciences, NJ, USA) were used to visualize using the ChemiDoc™ XRS Imaging Systems and Software (Bio-Rad, Biosciences, USA). The band intensities of the blots were analysed using Scion Image software (Scion Corporation).

### 3.1. Statistical Analysis

The values are expressed as mean ± SEM. Contractile responses are expressed as % of contraction induced by 75 mM KCl in the same aortic ring. The Gaussian distribution of the variables was previously analyzed using the D'Agostino–Pearson omnibus normality test. Fitting concentration-response curves were constructed and analyzed using nonlinear regression analysis. Statistical comparisons between the different groups were performed by one- or two-way analysis of variance (ANOVA), followed by Bonferroni's post hoc test. Differences between means with a value of *p* < 0.05 were considered statistically significant (statistical software: Prism 7.0, GraphPad Software Inc.).

## 4. Results

### 4.1. CCEC Changing the Interval Time between Angiotensin II Concentrations

**Figure 1:**
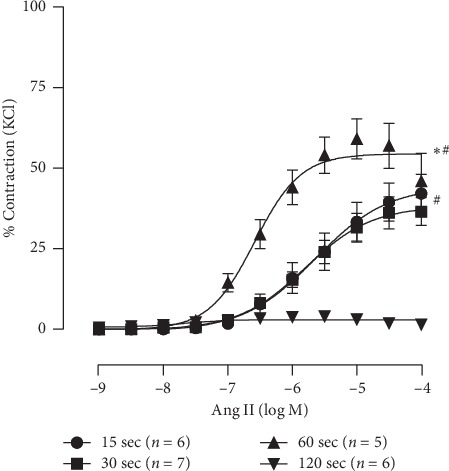
Cumulative concentration-effect curves (CCEC) with different intervals between angiotensin II applications. Data are expressed as mean ± SEM of maximum KCl contraction induced. ^*∗*^*p* < 0.05 vs. 30 sec and 15 sec; ^#^*p* < 0.05 vs. 120 sec.

We first evaluated the optimal interval time between angiotensin II concentrations in the CCEC. Intervals of 15, 30, 60, and 120 seconds were applied between each angiotensin II concentration.  Figure 1 summarizes the contractile response Ang II induced in aortic rings in each time interval. The 60-second interval exhibited an enhanced contractile response when compared with other intervals as indicated by the two main pharmacodynamic parameters of the concentration-response curve. Applying each concentration every 60 seconds reached the highest Rmax (15 sec, 44 ± 5%; 30 sec, 38 ± 3%; 60 sec, 54 ± 3%; 120 sec, 3 ± 1%, Figure 1) as well as the highest sensitivity (pEC_50_: 15 sec, 5.6 ± 0.2 M; 30 sec, 5.8 ± 0.1 M; 60 sec, 6.6 ± 0.1 M; 120 sec, none; −log M Ang II). Responsiveness to Ang II was not observed when applied with 120 seconds of interval.

### 4.2. NCCEC versus CCEC to Ang II

**Figure 2:**
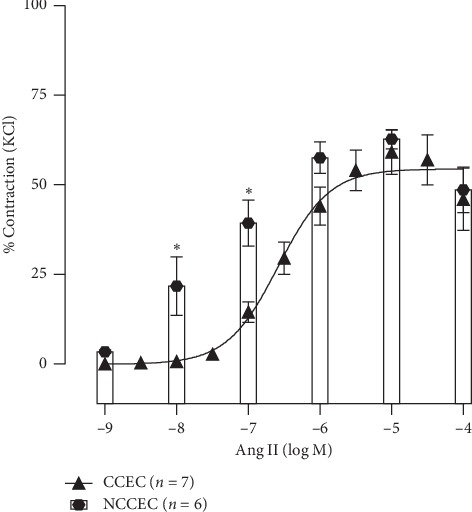
Interpolation of cumulative (CCEC) and noncumulative concentration-response curves (NCCEC) to angiotensin II (Ang II). Data are expressed as mean ± SEM. ^*∗*^*p* < 0.05 vs. CCEC.

The maximal response obtained in the CCEC versus the NCCEC protocol was also compared.  Figure 2 shows no difference in the maximal response between these two methods (CCEC, 56 ± 3% vs NCCEC, 60.0 ± 3% of KCl contraction). However, higher contraction was developed in the NCCEC protocol at smaller concentrations (at 10^−8^M: CCEC, 0 ± 3% vs NCCEC, 21 ± 8%; at 10^−7^M: CCEC 12 ± 2% vs NCCEC, 39 ± 6%, *p* < 0.05).

### 4.3. Effect of Repetitive Vascular Reactivity Experiments

**Figure 3:**
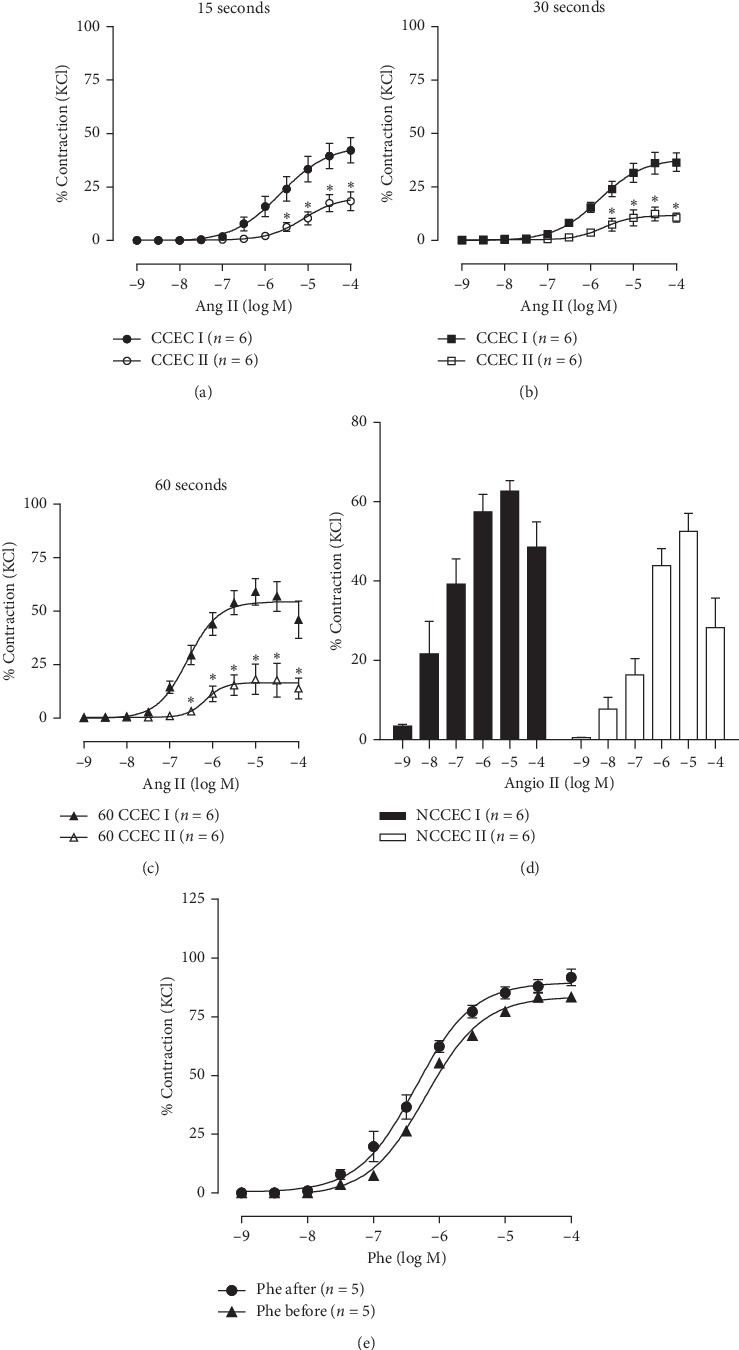
Attempt to use the same aortic tissue for two experiments. (a) CCEC I and CCEC II with 15-second interval between each angiotensin II application, (b) CCEC I and CCEC II with 30-second interval, (c) CCEC I and CCEC II with 60-second interval, (d) NCCEC I and NCCEC II to Ang II, and (e) CCEC I and CCEC II to phenylephrine. For all cases, the second experiment was performed 60 min after the first experiment with each tissue. Data are expressed as mean ± SEM.

We also evaluated the possibility of performing a feasible second CCEC or a second NCCEC to Ang II using the same aortic rings (CCEC II and NCCEC II). Because no contraction was observed in the CCEC I within 120-second interval (Figure 1), CCEC II for this interval was not performed. Figures 3(a)–3(c) summarize the results of Ang II-induced contractions of aortic rings in the CCEC I and CCEC II. Aortic rings of CCEC II exhibited a decreased maximal contractile response to Ang II when compared to CCEC I. The data show a significant decrease in *R*_max_ (15 sec, −51%; 30 seconds, −57%; 60 seconds, −74%).  Figure 3(d) shows the results for NCCEC I and II. There seems to be a lower impairment in the NCCEC protocols versus the CCEC protocols. There was only a 17% decrease in the maximal contraction response in the NCCEC I versus NCCEC II. Finally, to prove that this pattern of response is specific to Ang II, vascular reactivity was also performed with adrenergic *α*1-agonist phenylephrine, and results showed no impairment in the CCEC II versus CCEC I (Figure 3(e)).

### 4.4. Effect of Resting Tension

**Figure 4:**
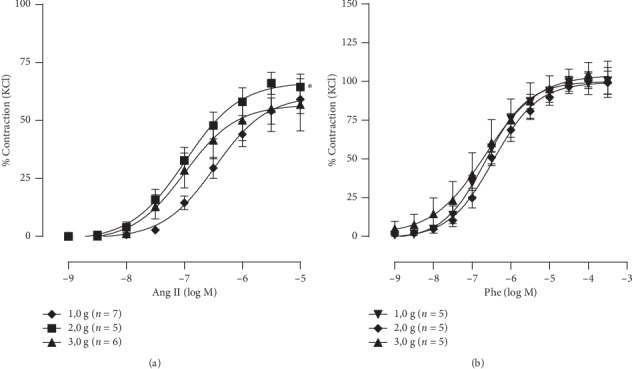
Cumulative concentration-response curves at different resting tension to (a) angiotensin II (Ang II) and (b) phenylephrine (Phe). Data are expressed as mean ± SEM. ^*∗*^*p* < 0.05 vs. 1 g and 3 g.

We also tested the influence of resting tension in the vascular response to Ang II.  Figure 4(a) compares different resting tensions. The response at 2 g (*R*_max_: 69.7 ± 4%; pEC_50_ : 6.92 ± 0.13–logM) of resting tension of aortic rings did modify the sensitivity and the maximal response to Ang II when compared with 1g (*R*_max_: 64.9 ± 5%; pEC_50_: 6.45 ± 0.07−logM) and 3 g (*R*_max_: 64.8 ± 4%; pEC_50_: 6.86 ± 0.08−logM).

To prove that this response is specific to Ang II, CCEC to phenylephrine was also performed at resting tension of 1 g, 2 g, and 3 g, and difference was not observed in the maximal response or in the sensitivity to phenylephrine using different resting tensions (Figure 4(b)). These data suggest that the resting tension significantly contributes to the vascular responsiveness to Ang II.

### 4.5. Role of AT1 *β*-Arrestin-Biased Signaling

**Figure 5:**
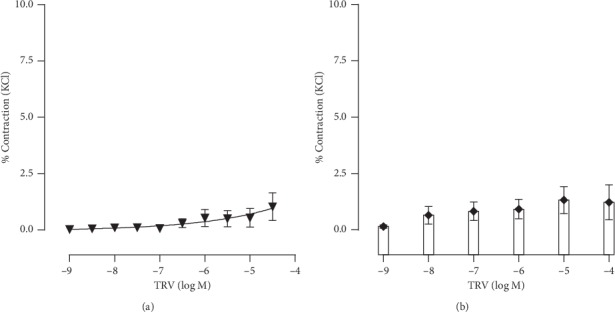
Vascular reactivity to TRV023. (a) Cumulative (CCEC) and (b) noncumulative concentration-response curves (NCCEC) to TRV023 (TRV). Data are expressed as mean ± SEM, *n* = 5 for each condition.

Next, we tested if the activation of *β*-arrestin through the AT1R would lead to vascular contraction. Both CCEC (Figure 5(a)) as well as NCCEC protocol were performed (Figure 5(a)) and showed no significant contraction to TRV023. These data suggest Ang II-induced vascular contraction is G-protein dependent and *β*-arrestin independent.

**Figure 6:**
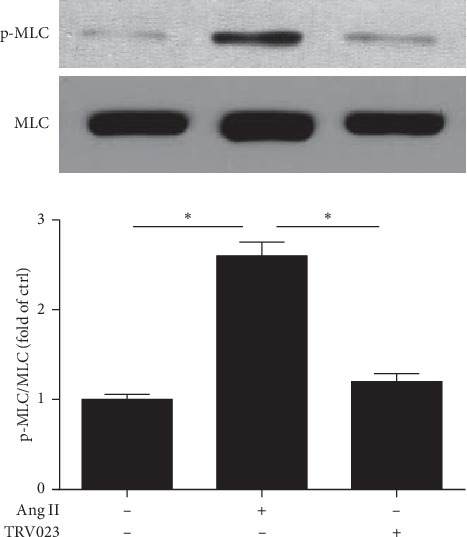
Immunoblot of proteins extracted from aortic rings stimulated with angiotensin II (1 *µ*M) or TRV023 (1 *µ*M) to myosin light chain phosphorylation of endothelium-denuded aortic rings. Representative western blot and p-MLC/total MLC ratio. Data are expressed as mean ± SEM. *n* = 4. ^*∗*^*p* < 0.05.

To further exploit the *β*-arrestin independent pathway in Ang II-induced vascular contraction, myosin light chain phosphorylation (MLC) was also analyzed Figure 6 shows that only Ang II, but not TRV023, induces p-MLC phosphorylation in aortic rings.

## 5. Discussion

Here, we have studied several parameters in the vascular reactivity assay. We have observed that 60 seconds was the optimal interval time between each concentration of angiotensin II. We also observed that 2 g was the best resting tension and that performing 2 vascular reactivity curves is not feasible. Finally, we observed that building a curve with NCCEC concentrations of angiotensin II is also possible and does not interfere in Rmax or pEC_50_.

At first, we showed the vascular contraction using cumulative concentration-response curves to angiotensin II in rat aortic rings according to the time between each application of the drug and observed that Ang II effects were ideal with approximately 60 seconds between each Ang II application. Shorter times (15 or 30 s) were unable to induce whole contraction, as indicated by lower potency expressed by pD2 and lower *R*_max_. On the other hand, curves with longer duration between each Ang II application did not show contraction, even at low Ang II concentrations. This may be due to the desensitization of the AT1R, showing that the vascular contractions to angiotensin II depends more on the time between Ang II concentrations than on the concentration of Ang II applied.

Next, we showed that the noncumulative Ang II curve develops more contractile force at low concentrations than a cumulative curve. We also demonstrated in our study that regardless of whether single concentration or cumulative concentration-response experiments are performed, the vascular response to angiotensin is lower in the second experiment, but this impairment was much lower in single concentration compared to concentration response demonstrating that single concentration may be the most indicated when it is necessary to perform two curves in the same ring. Linder et al. showed that this blunted effect in the CCEC II occurs due to Ang II-induced tachyphylactic contractile responses in aortic rings [12].

It has already been published by our team [19, 20] and others [21–24] the mechanosensitive properties of AT1R in the cardiovascular system. In this study, we showed that *ex vivo* stretch of aortic rings changes the vascular reactivity to Ang II. Basal tension of 2 g had higher EC50 as well as Rmax when compared to 1 g of basal tension. In 2010, Liu et al. [25] showed that mechanical stretch potentiates Ang II-induced proliferation of smooth muscle cell *in vitro*. In 2014, Tang et al. [26] showed the allosteric modulation of AT1R signaling by membrane stretch. They showed different activation of ERK1/2 by Ang II depending on the stretch that the cells were submitted, suggesting higher sensitive to Ang II as higher initial cell stretching. In 2016, Abraham et al. [27] found that mice lacking AT1R were unable to produce a Frank–Starling force in response to changes in cardiac volume, which reveals that the AT1R signaling pathway is vital in the mechanotransduction of the heart. However, we are the first to show in the functionally vascular system the AT1R mechanical properties. This may be of importance in disease conditions such as hypertension or stroke where RAS blockers may have a role even without significant increase in the concentration of Ang II [28, 29].

Besides the standardization of the vascular reactive protocol to Ang II, we also aimed to study the effect of the biased ligand of the AT1R (TRV023) to induce vasoconstriction, similarly to Ang II. We and others have already studied its effects on the renal [7] and cardiac system [5–8]; however, this is the first study to show its direct effects in the vasculature. We have showed that, although TRV023 also binds to AT1R, it did not induce vasoconstriction nor phosphorylation of MLC2, demonstrating that the vasoconstrictor effect of Ang II is G-protein mediated. We have not observed direct vasodilatatory effects of TRV023, but we have used endothelium-free aorta and the vessel was not preconstricted. Future studies should perform these in the presence of the endothelium and in preconstricted vessels to address these issues. TRV027 (and TRV023) is a “biased” ligand of the AT1R, selectively antagonizing the negative effects of angiotensin II, while preserving the potential procontractility effects of AT1R stimulation. After several positive results in the cellular and rodent models, researchers started the BLAST-AHF (Biased Ligand of the Angiotensin Receptor Study in Acute Heart Failure), which was designed to determine the safety, efficacy, and optimal concentration of TRV027 to advance future studies. However, after 621 patients enrolled, this phase IIb dose-ranging study did not improve clinical status after a 30-day follow-up [8, 30]. Here, we showed that the vasoconstrictor effector of AT1R is G-protein dependent and more importantly that TRV does not have vasoconstrictor effects.

## Data Availability

The data used to support the findings of this study are available from the corresponding author upon request.
